# COVID-19: positive experience with differentiated tactics of mechanical ventilation of the lungs for different phenotypes (L-phenotype)

**DOI:** 10.1186/s41231-022-00122-8

**Published:** 2022-07-08

**Authors:** Valery Piacherski, Lidziya Muzyka, Dzyanis Zhylynski

**Affiliations:** Department of Anesthesiology and Intensive Care, Mogilev Regional Clinical Hospital, 212026 A. Kuleshov str., 3-36, Mogilev, Republic of Belarus

**Keywords:** CoVID-19 pneumonia, Acute Respiratory Distress Syndrome (ARDS), Type L, Mechanical ventilation of the lungs

## Abstract

**Relevance:**

Studies have previously been published on a possible differential approach to respiratory therapy in patients with COVID-19 depending on the L- or H-phenotype.

The authors believe that early tracheal intubation reduces the risk of lung injury. The use of deep sedation and low PEEP (6–8 cmH_2_O) and early intubation may prevent transition to type H.

**Method and results:**

Eleven patients with COVID-19 type L pneumonia received respiratory support based on the proposed guidelines. Eight women and three men (ages 45 to 84) with COVID-19 type L pneumonia were treated in the intensive care unit. Did they all receive oxygen therapy up to 15 L/min. high-flow oxygen therapy up to 60 L/ min, non-invasive ventilation of the lungs. If it was impossible to reduce FiO_2_ from 100 to 75% within 2–3 h or if the patient was intolerant to NIV, early tracheal intubation was used.

The minute ventilation volume was set to maintain CO_2_ <60 mmHg. and pH>7.25 in venous blood. Sedation was performed by intravenous titration of fentanyl and propofol. If deeper sedation was required to synchronize the patient to the ventilator, intravenous muscle relaxants were used over 24-48 hours (bolus or intravenous titration) instead of sedation.

**Conclusion:**

All 11 patients were successfully weaned from the mechanical ventilation of the lungs. A differentiated approach to respiratory therapy for COVID-19 L-type pneumonia proved to be an effective approach in these patients.

It is probably worth avoiding deep sedation of patients on mechanical ventilation with L-type pneumonia, which would reduce the time spent on mechanical ventilation and reduce the risk of mortality from nosocomial bacterial infection.

The new MVL strategy for L-type pneumonia and the problem of deep sedation require more research. But the available data suggests that it probably has benefits.

## Relevance

In 2020 Gattinoni et al. published a letter noting that ARDS in COVID-19 often does not correspond to the notion of classical ARDS [1]. It was noted that very severe hypoxemia often maintains high respiratory compliance (> 50 cmH_2_O) and low perfusion [2, 3]. This type is L (or type 1) lung damage.

Then Gattinoni et al. published a paper on a possible differentiated approach to respiratory therapy of patients with COVID-19 depending on the L or H phenotype [4].

The authors consider early tracheal intubation to reduce the risk of lung damage [4]. The use of deep sedation and low PEEP (6–8 cmH_2_O). Early intubation can prevent transition to type-H [3].

## Method and results

We report our experience with these recommendations in 11 patients with COVID-19 type L pneumonia. These patients received respiratory support based on the proposed recommendations [3, 4]. Eight women and three men (ages 45 to 84) with COVID-19 type-L pneumonia were treated in the intensive care unit (October-November 2021). They all received oxygen therapy up to 15 L/min. If hypoxemia increased, high-flow oxygen therapy up to 60 L/min was administered. If it was ineffective, noninvasive lung ventilation was established. If it was not possible to reduce FiO_2_ from 100 to 75% within 2 to 3 h, or if the patient was intolerant to NIV, we used early tracheal intubation.

The minute ventilation volume was set to maintain CO_2_ < 60 mmHg and pH > 7.25 in venous blood (within the protective MVL). Sedation was performed by intravenous titration of fentanyl and propofol. If deeper sedation was required to synchronize the patient with the ventilator, we used intravenous muscle relaxants for 24–48 h (bolus or intravenous titration) instead of sedatives. We used relaxants in these cases, so as not to increase the dosage of sedatives and not to prolong the stay on MVL due to prolonged withdrawal from sedation (if laboratory indications for weaning from the MVL apparatus appear).

When a trend appeared to reduce the minimum required FiO_2_ to 75%, we reduced the sedation and sought to wean the patient off mechanical ventilation of the lungs (MVL) as quickly as possible. On average, by day 14, patients were weaned from the MVL. They were extubated at FiO_2_ levels of 45–50%. Immediately after extubation, high-flow oxygen therapy was established with a flow of 40 L/min in the prone position. One woman spent 8 weeks on the ventilator and was also weaned from the machine.

Pron-positioning in these patients slightly increased saturation during ventilation, but we used it to improve sputum drainage. After extubation, prone-position had a significant effect on the increase in oxygen saturation in all 11 patients.

## Discussion and conclusion

A differentiated approach to respiratory therapy for L-type COVID-19 pneumonia was an effective approach in these patients.

As one of the causes of lethality of COVID-19 patients on the ventilator is the accession of secondary nosocomial bacterial infection, we did not use deep sedation in order to wean the patient off the ventilator as soon as possible in case of positive disease dynamics. Morphine and diazepam were not used in order to reduce the time the patient spent on the ventilator. As the long-term use of these drugs prolongs the time of ventilatory ventilation. It is probably worth avoiding deep sedation of patients on the ventilator with L-type pneumonia, which, would reduce the patient’s stay on the ventilator and reduce the risks of mortality from hospital-acquired bacterial infection.

A new MVL strategy for L-type pneumonia and the problem of deep sedation need more research. But existing data suggests that it has advantages.

## Data Availability

Available upon reasonable request by e-mail.
